# Dendritic Cell-Mediated-Immunization with Xenogenic PrP and Adenoviral Vectors Breaks Tolerance and Prolongs Mice Survival against Experimental Scrapie

**DOI:** 10.1371/journal.pone.0004917

**Published:** 2009-03-19

**Authors:** Martine Bruley Rosset, Antoine Sacquin, Sylvie Lecollinet, Thomas Chaigneau, Micheline Adam, François Crespeau, Marc Eloit

**Affiliations:** 1 INSERM UMR 938, Paris, France; 2 UPMC Univ Paris 06, Hôpital Saint-Antoine, Saint-Antoine, Paris, France; 3 UMR1161 Virologie, INRA, AFSSA, ENVA, Ecole Nationale Vétérinaire d'Alfort, Maisons Alfort, France; 4 Laboratoire d'anatomopathologie, Ecole Nationale Vétérinaire d'Alfort, Maisons Alfort, France; University of Edinburgh, United Kingdom

## Abstract

In prion diseases, PrP^c^, a widely expressed protein, is transformed into a pathogenic form called PrP^Sc^, which is in itself infectious. Antibodies directed against PrP^c^ have been shown to inhibit PrP^c^ to PrP^Sc^ conversion in vitro and protect in vivo from disease. Other effectors with potential to eliminate PrPSc-producing cells are cytotoxic T cells directed against PrP-derived peptides but their ability to protect or to induce deleterious autoimmune reactions is not known. The natural tolerance to PrP^c^ makes difficult to raise efficient adaptive responses. To break tolerance, adenovirus (Ad) encoding human PrP (hPrP) or control Ad were administered to wild-type mice by direct injection or by transfer of Ad-transduced dendritic cells (DCs). Control Ad-transduced DCs from Tg650 mice overexpressing hPrP were also used for immunization. DC-mediated but not direct administration of AdhPrP elicited antibodies that bound to murine native PrP^c^. Frequencies of PrP-specific IFNγ-secreting T cells were low and in vivo lytic activity only targeted cells strongly expressing hPrP. Immunohistochemical analysis revealed that CD3^+^ T cell infiltration was similar in the brain of vaccinated and unvaccinated 139A-infected mice suggesting the absence of autoimmune reactions. Early splenic PrP^Sc^ replication was strongly inhibited ten weeks post infection and mean survival time prolonged from 209 days in untreated 139A-infected mice to 246 days in mice vaccinated with DCs expressing the hPrP. The efficacy appeared to be associated with antibody but not with cytotoxic cell-mediated PrP-specific responses.

## Introduction

In prion diseases, cellular prion protein (PrP^c^), a normal host protein present in the majority of tissues and highly expressed in the nervous system [1], is converted into a protease resistant form (PrPres), also called scrapie PrP (PrP^Sc^), which is in itself infectious [2]. PrP^c^ is a protease sensitive glycoprotein which is attached to the cell membrane by a glycophosphatidylinositol anchor. PrP^Sc^ has a high β-sheet content and prone to aggregation. In the most widely accepted model, PrP^Sc^ interacts with PrPc and converts it into PrP^Sc^ [3]. Prion diseases are characterized by long asymptomatic periods of incubation. Yet, it has been reported that when PrP^Sc^ accumulation is stopped, PrP^Sc^ can be cleared, injury is reduced by compensatory neuronal mechanisms and synaptic function is restored [4], paving the way to therapeutics aimed at blocking PrP^Sc^ accumulation.

Obtaining an effective prophylactic or therapeutic vaccines against prion diseases combines the difficulties of developing vaccines against persistent infections, and those inherent to immunotherapy against self-antigens. Moreover, it remains possible that protection against challenge versus to cure established disease might involve different effectors. There is now good evidence showing that anti-PrP antibodies are able to inhibit PrP^c^ to PrP^Sc^conversion in vitro [5]–[8] and to protect mice against prion disease in vivo providing they recognize native PrP^c^ [9]–[12]. While the mechanism underlying their therapeutic effect has not yet been elucidated one explanation is that antibodies alter PrP trafficking and thereby inhibit the PrP^c^ to PrP^Sc^ conversion process [3]. However, it has been suggested that the main role of antibodies was to diminish the pool of GPI-anchored PrP^c^, thus leading to protection or to a delay in animal death [13]. Opsonisation of PrPSc by microglial cells may also be involved.

Other effectors with potential to eliminate PrP^Sc^-producing cells are cytotoxic T cells (CTLs) directed against PrP-derived peptides but the repertoire of CD8^+^ T cells in wild-type (wt) mice and their ability to protect from prion disease or to induce deleterious autoimmune reactions have never been addressed. In prion infected mice, cells that replicate and accumulate PrP^Sc^ are follicular dendritic cells (FDC) [14] while dendritic cells (DCs) uptake and transport PrP^Sc^ [15] and participate in the process of neuroinvasion [16]. Both cells are MHC class I positive; FDC express naturally high level of PrP^c^ that became much stronger after immune stimulation [17] and DCs upregulate its expression upon in vivo maturation [18] and therefore might be target of CTL. On the other hand, it remains possible that PrP^Sc^ accumulation could impair proteasome function and peptide presentation by MHC I [19].

In addition to the above difficulties in identifing relevant immune effectors, mechanisms maintaining peripheral tolerance to self-PrP [20] present a major obstacle to the development of vaccines with the risk of autoimmune diseases in cases of success. We have previously shown that T cell tolerance can be overcome in wt mice after PrP peptide immunization with oligo-CpG. However, regarding B cell tolerance, the antibodies raised by this protocol recognized recombinant but not native PrP^c^ [21]–[22]. B cells specific for PrP epitopes exposed at the cell surface in the native conformation of PrP^c^ were presumably deleted or anergized in PrP expressing mice, and antibodies recognized only epitopes buried in the PrP^c^ molecule [23]. These treatments were shown to delay only moderately prion disease progression [24]. Despite these observations, good evidence now exists that the anti-PrP antibody repertoire is not totally deleted but that strong immunogen delivery is necessary to break tolerance. PrP displayed on retroviral particles induced humoral responses to self-native PrP^c^ in wt mice [25]. In another report, a peptide containing a B cell epitope (PrP144-152) of the murine PrP (mPrP) was inserted into the L1 major capsid protein of the bovine papilloma virus and used to immunize rabbits. These papillomavirus-like particles were reported to induce autoantibodies that inhibited de novo synthesis of PrP^Sc^ in cell culture [26]. Xenogenic antigens can, in some instances, overcome tolerance to autoantigens. PrP is highly conserved in mammals, among which 90% of the aminoacid sequence is shared. Nevertheless vaccination with bovine PrP has been shown to elicit antibodies that cross react with mPrP [27].

T epitopes can also be targeted by using xenoantigens: immunization with human HER-2/neu peptide enhanced immunity to a self-HER-2/neu CTL epitope and efficiently protected against tumor growth [28]. Administration of recombinant vaccinia virus encoding the human but not mouse homologue (differing in the three HH-2-terminal aminoacids) of gp100 melanoma antigen to mice elicited a specific CD8^+^ T cell response and treated established B16 melanoma [29].

Tolerance in the PrP-specific CD4^+^ Th compartment is likely to be responsible for immune unresponsiveness. Thus to improve the immunization procedure, we inserted human PrP (hPrP) into an adenovirus vector that brings foreign Th specific for capsid proteins and may be potent in generating antibody CD4^+^ and CD8^+^ CTL responses against both the viral antigens and the transgene [30]. To induce a potent immune response, prime-boost vaccination has been used in view of the high immunogenicity of this strategy, as demonstrated in several experimental models [31]–[33].

We show here that delivery to C57BL/6 (B6) wt mice of DCs transduced with xenogenic PrP (i.e. human) encoded by an adenoviral (Ad) vector is able to break immune B-cell tolerance against the endogenous mPrP. The production of high levels of antibody directed against the native conformation of mPrP resulted in prion disease attenuation without triggering T cells, thus avoiding autoimmune reactions.

## Results

### Immunization of wt mice with AdhPrP induces weak immune responses against murine PrP and little increase in survival time after scrapie challenge

**Figure 1:**
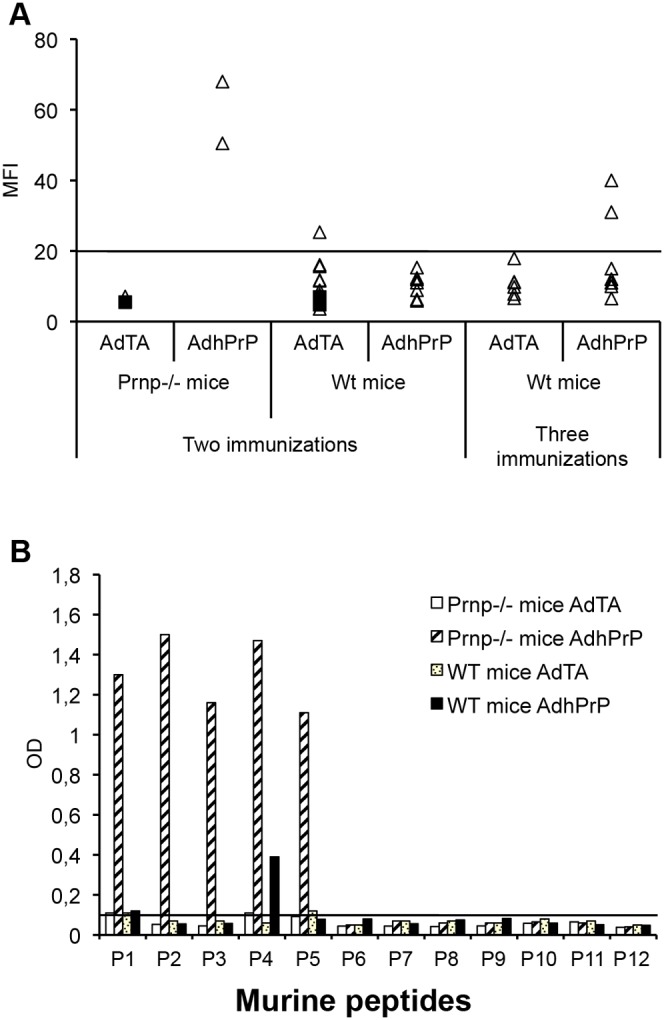
Antibody response against native PrPc elicited in *Prnp^−/−^* and C57BL/6 wt mice after direct Ad immunization. (A). Sera collected after two or three immunizations with AdTA or AdhPrP were tested (1/50 dilution) for their capacity to bind mPrP expressed on activated EL4 cells. The level of serum binding was expressed as mean fluorescence intensity (MFI) and revealed by incubation with a biotinylated anti-Ig followed by APC-conjugated streptavidin. Fluorescence was analysed by flow cytometry. Each point represents the MFI of an individual serum sample from immunized (triangle) or unimmunized (black square) mice. (B). Epitope mapping of serum antibodies generated after immunization. Individual immune sera (1/50 dilution) were tested by ELISA for binding to each of mPrP peptides (10 µg/well) coated on plastic microplates.

We demonstrated in a previous study that cells transduced with AdhPrP express a high level of hPrP, as shown by Western blotting [34]. To generate an anti-PrP-specific immune response, priming with AdhPrP and boosting with phPrP was performed. This regimen was able to induce antibodies to native hPrP in both *Prnp^−/−^* and C57BL/6 wt mice (Data S1). Following immunization of B6 wt mice, sera were collected 2 weeks after the prime-boost and tested by indirect immunofluorescence for the presence of antibodies binding to mPrP on EL4 cells (Fig. 1A). After immunization with AdhPrP but not with AdTA, *Prnp^−/−^* mice developed an humoral response to native mPrP validating Ad vectors as efficient immunogens. In wt mice, the level of the humoral response for mPrP was nearly absent (Fig. 1A). A second boost was performed 3 weeks after 139A challenge and raised substantial amount of antibodies recognizing EL4 cells in only 2/7 wt mice. The epitope specificities recognized by serum antibodies were identified by ELISA using libraries of overlapping mPrP peptides (Table 1). Sera from *Prnp^−/−^* mice bound to the N-terminal portion of mPrP between aminoacids (aa) 29–127) while sera of wt mice recognized only P4 (PrP83-112) (Fig. 1B).

**Table 1:** Comparative aminoacid sequences of overlapping peptides derived from murine PrP.

Murine Peptides	Aminoacid Sequences*																													
23–52: P1	K	K	R	P	K	P	G	G	W	N	T	G	G	S	R	Y	P	G	Q	G	S	P	G	G	N	R	Y	P	P	
39–67: P2	P	G	Q	G	S	P	G	G	N	R	Y	P	P	Q	G	G	**–**	**T**	W	G	Q	P	H	G	G	G	W	G	Q	P
68–97: P3	H	G	G	**S**	W	G	Q	P	H	G	G	**S**	W	G	Q	P	H	G	G	G	W	G	Q	G	G	G	T	H	**N**	Q
83–112: P4	P	H	G	G	G	W	G	Q	G	G	G	T	H	**N**	Q	W	N	K	P	S	K	P	K	T	N	**L**	K	H	**V**	A
98–127: P5	W	N	K	P	S	K	P	K	T	N	**L**	K	H	**V**	A	G	A	A	A	A	G	A	V	V	G	G	L	G	G	Y
118–142: P6	G	A	V	V	G	G	L	G	G	Y	M	L	G	S	A	M	S	R	P	**M**	I	H	F	G	N					
128–157: P7	M	L	G	S	A	M	S	R	P	**M**	I	H	F	G	**N**	D	**W**	E	D	R	Y	Y	R	E	N	M	**Y**	R	Y	P
143–172: P8	D	**W**	E	D	R	Y	Y	R	E	N	M	**Y**	R	Y	P	N	Q	V	Y	Y	R	P	**V**	D	**Q**	Y	S	N	Q	N
158–187: P9	N	Q	V	Y	Y	R	P	**V**	D	**Q**	Y	S	N	Q	N	N	F	V	H	D	C	V	N	I	T	I	K	Q	H	T
173–189: P10	N	F	V	H	D	C	V	N	I	T	I	K	Q	H	T	V	T													
193–218: P11	K	G	E	N	F	T	E	T	D	V	K	M	M	E	R	V	V	E	Q	M	C	**V**	T	Q	Y	**Q**	**K**			
212–232: P12	M	C	V	T	Q	Y	**Q**	**K**	E	S	Q	A	Y	Y	**D**	**G**	**R**	R	**S**	S	**S**									

* Bold characters represent the aminoacids that differ between murine and human PrP peptide sequences.

**Figure 2:**
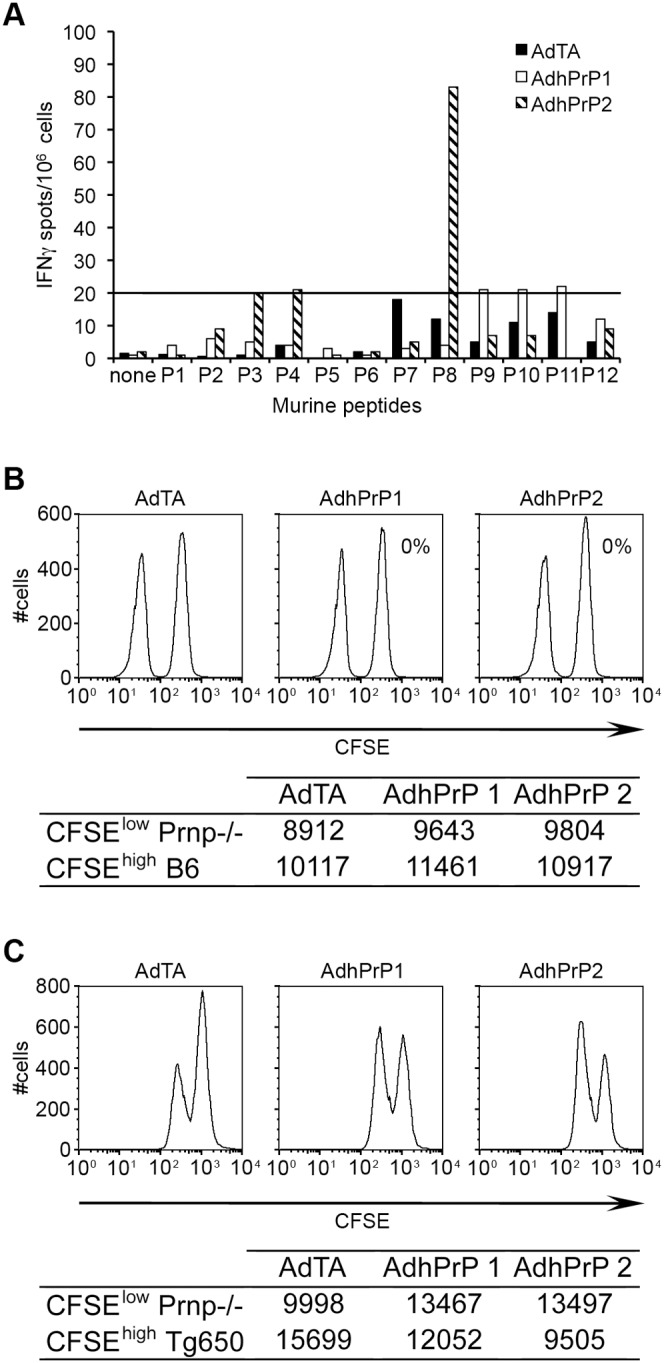
T cell responses in vaccinated C57BL/6 wt mice. (A). Frequency of IFN-γ secreting T cells in the spleens of immunized mice evaluated by a ELISPOT assay. Spleen cells (10^6^/well) from two individual mice receiving AdTA or AdhPrP were stimulated 18 h at 37°C with each of 30-mer peptides from mPrP at a concentration of 10 µg/ml. The frequency of peptide-specific IFN-γ secreting T cells was calculated after substracting the mean number of spots obtained in the absence of peptide. Each column shows the mean numbers of spots±SD per 1×10^6^ spleen cells of triplicate cultures. (B–C). In vivo cytotoxic activity was measured by flow cytometry: spleen cells from mice vaccinated with AdTA or AdhPrP were collected 20 h after injection of 10^7^ CFSE^low^ *Prnp^−/−^* cells mixed with 10^7^ CFSE^high^ cells expressing either (B) mPrP (C57BL/6) or (C) hPrP (Tg650). Number of CFSE^low^ and CFSE^high^ cells in the spleen of individual immunized mice and percentage of specific lysis of PrP+ over *Prnp^−/−^* splenocytes were calculated as detailed in “Materials and Methods”.

The T cell response was evaluated by measuring by ELISPOT the precursor frequency of IFNγ-secreting spleen cells upon stimulation with the library of murine PrP peptides (Table 1). Figure 2A shows that only one mouse out of two responded and that the PrP-specific T cell response was directed against one epitope lying in the murine P8 region. The T cell response to hPrP peptides was weak (data not shown).

The in vivo lytic capacity of immunized mice was measured by injecting CFSE*^high^* C57BL/6 or Tg650 cells mixed with CFSE*^low^ Prnp^−/−^* cells. AdhPrP-immunized mice were able to lyse 23% or 33% of target cells expressing hPrP, but unable to lyse cells expressing mPrP (Fig. 2B). AdTA immunized-mice did not display any cytotoxic activity. Thus, immunization with AdhPrP induced an in vivo cytotoxic activity for hPrP overexpressing cells. These results were confirmed by the absence of in vitro cytotoxic activity for B6 targets in AdTA- or AdhPrP-vaccinated wt mice (data not shown).

**Figure 3:**
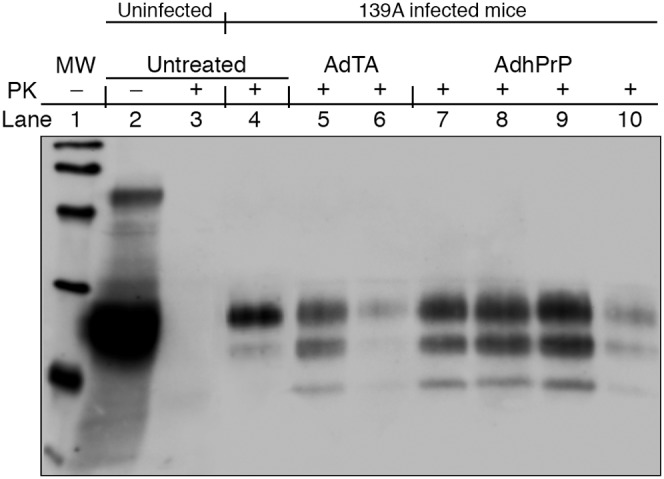
Measurement of PK-resistant PrPres in untreated and AdTA- or AdhPrP-treated C57BL/6 wt mice. Western blotting was performed on proteinase K (PK)-treated PTA precipitates prepared from 10 mg of spleens collected at 10 weeks after challenge with 0.5% 139A scrapie. Samples were digested with 50 µg/ml PK, heated at 100C° and loaded on 12.5% polyacrylamide/SDS gel. Blots were incubated with 1/5000 diluted SAF84 (Anti-mPrP mAb) and revealed with peroxidase-conjugated goat anti-mouse Ig antibody. A. Lane 1 = Molecular weight. Lane 2 = normal spleen. Lane 3 = normal spleen+PK. Lanes 4 = 139A-infected spleen+PK. Lanes 5–6 = AdTA-treated infected spleen+PK. Lanes 7–10 = AdPrP-treated infected spleen+PK.

The effect of vaccination on the outcome of experimental scrapie was examined after challenging wt mice i.p. with a 0.5% 139A brain homogenate 2 weeks after the prime-boost. Protection against early PrP^Sc^ invasion was evaluated in the spleen of mice collected 10 weeks post-infection (pi) by measuring the amount of PK-resistant PrP (PrPres) by Western blot analysis of PTA homogenates (Fig. 3). The 23–27 Kd band corresponding to endogenous PrP^c^ prepared from normal spleen homogenate (Lane 2) was eliminated after PK treatment (Lane 3) confirming the known sensitivity of PrP^c^ to proteases. Homogenates prepared from a spleen from 139A-infected wt mice was resistant to PK treatment. An inhibition of PrPres accumulation was observed in one of two AdTA-treated mice (Lanes 5–6) and one of four AdhPrP-treated mice (Lanes 7–10).

To quantify more precisely the effect of the different vaccination protocols on the disease, we first studied the survival of control mice as a function of the infectious dose of 139A scrapie homogenates administered to C57BL/6 mice Table 2A showed that mice receiving 0.5% 139A succumbed to the disease at 209±8 dpi and that a 10- fold (0.05%) or 50- fold (0.01%) dilution of the 139A homogenate prolonged the mean survival of 20 and 33 days, respectively. A low but significant delay of 13 and 22 days was obtained in AdTA- and AdhPrP-vaccinated mice compared to control mice challenged with 0.5% homogenate (p = 0.002 and p<0.0001, respectively;Table 2B). The mean incubation time and the duration of the clinical stage were also longer in mice immunized with AdhPrP than in untreated controls (52±6 vs 41±8 days, p = 0.007).

**Table 2:** Comparison of incubation times, survival and clinical stage durations between non-immunized infected wt mice (A) or wt mice immunized either (B) directly with AdTA or AdhPrP or (C) after transduction into DCs derived from C57BL/6 or Tg650 mice.

Treatment	139A dose (100 µL)	Incubation time	Survival time		Clinical stage duration
		mean dpi±SD	individual scores (dpi)	mean dpi±SD	mean dpi±SD
**A-Dose Effect** a					
Untreated	0,5%	168±3	195-199-200-204-209-212-213-214-214-215-216-221	209±8	41±8
	0,05%	177±4	217-219-235-235-238	229±10	51±6
	0,01%	196±4	233-237-241-245-253-255	244±9	47±9
**B-Direct Injection** a					
AdTA	0,5%	175±5	218-220-221-221-226-226	222±3	47±7
		p^c^ = 0.01		p^c^ = 0.025	NS^c^
AdhPrP	0,5%	177±6	221-221-223-224-226-231-231-236-236-236-240-249	231±9	52±6
		p^c^ = 0.0005		p^c^ = 0.0001	p^c^ = 0.007
**C-DC-Transduced** b					
DCB6/AdTA	0,5%	179±10	215-217-217-232-236->300^d^	223±10	48±7
		p^c^ = 0.049		p^c^ = 0.008	NS^c^
DCB6/AdhPrP	0,5%	183±3	207-214-226-233-234-239	225±12	43±11
		p^c^ = 0.0001		p^c^ = 0.023	NS^c^
DCTg650/AdTA	0,5%	201±7	227-240-255-262->300^d^	246±16	44±13
		p^c^ = 0.0003		p^c^ = 0.001	NS^c^

a Mice were primed and boosted twice as described in Material and Methods and inoculated IP with 0.5% 139A brain homogenate two weeks after the second immunization. Results are presented in days post infection (dpi)±SD.

b Mice received three injections of 5×10^5^ DC as described in Material and Methods and were inoculated ip with 0.5% 139A brain homogenate one week after the second immunization. Results are presented in days post infection (dpi)±SD.

c Mann-Whitney Test; p significant compared to untreated mice: NS = non significant.

d One mouse survived longer than 300 dpi and no PrPres was detected by Western Blot in the spleen at time of sacrifice.

These results show that the direct administration of adenoviral vectors encoding a xenogenic PrP into wt mice induces only a marginal specific immunity against endogenous mPrP, showing that tolerance remained virtually intact, and a moderate prolongation of survival.

### Immunization of C57BL/6 mice with adenovirus-transduced DCs breaks immune tolerance and partially protects mice against challenge

**Figure 4:**
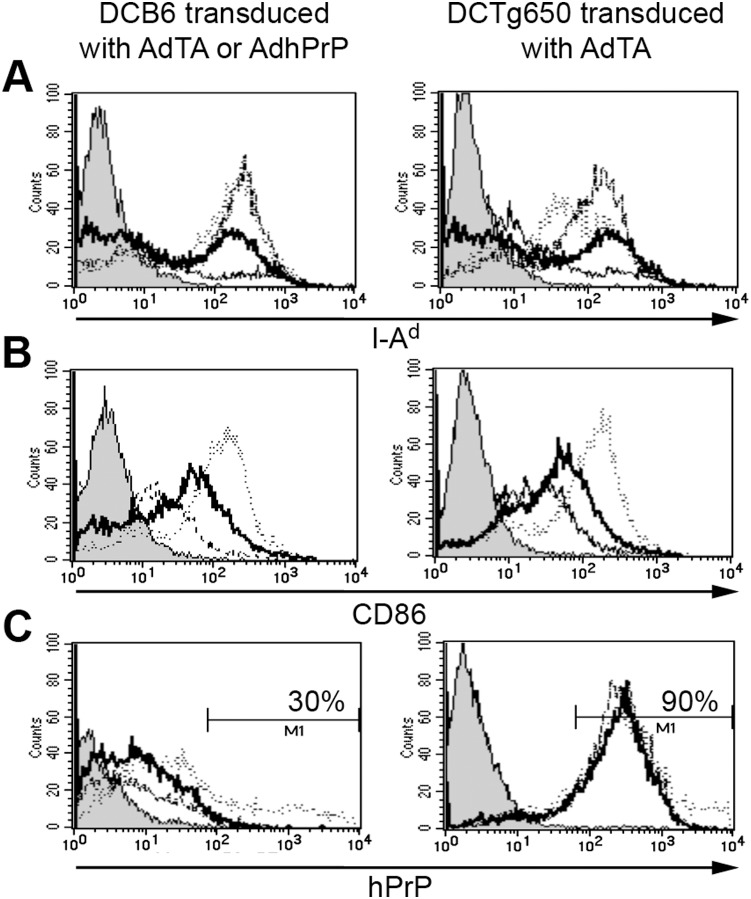
Expression of MHC class II, B7.2 and hPrP analysed by fow cytometry. BM-derived CD11c DCs prepared from C57BL/6 (left panel) and Tg650 (right panel) mice before (- - -) and after LPS maturation ( ) and AdTA (. _ . _) or AdhPrP (.....) transfection. Cell surface expression of IAd (a), CD86 (b) and hPrP (c) molecules. Staining controls (PE-conjugated anti-mouse IgG2a) are depicted in grey.

Immunization protocols can be based on Ad-transduced DCs that are potent stimulators of CTL responses [35]–[36] and of T-dependent antibody production [37]–[38]. In this assay, DCs from C57BL/6 and Tg650 (overexpressing hPrP) were prepared from bone marrow and maturated in the presence of GM-CSF and LPS. DCs from Tg650 mice were transduced with AdTA (DCTg650AdTA) and DCs from B6 mice with AdhPrP (DCB6AdhPrP) or AdTA alone (DCB6AdTA). Up regulation of class II (IAb) molecules was observed in CD11c^+^ DCs from B6 and Tg650 mice after maturation with LPS and was further amplified following transduction with AdTA or AdhPrP (Fig. 4a). Expression of B7.2 costimulatory molecule, detected with an anti-CD86 mAb, increased after LPS-induced maturation, and furthermore following transduction of DCs with AdhPrP (Fig. 4b). Transduction of DCs from B6 mice with AdhPrP but not AdTA induced a high expression of hPrP evidenced by 4F2 mAb staining in 30% of the CD11c^+^ DCs, while transduced and non- transduced CD11c^+^DCs from Tg650 mice expressed as expected a high level of hPrP in 100% of cells (Fig. 4c).

**Figure 5:**
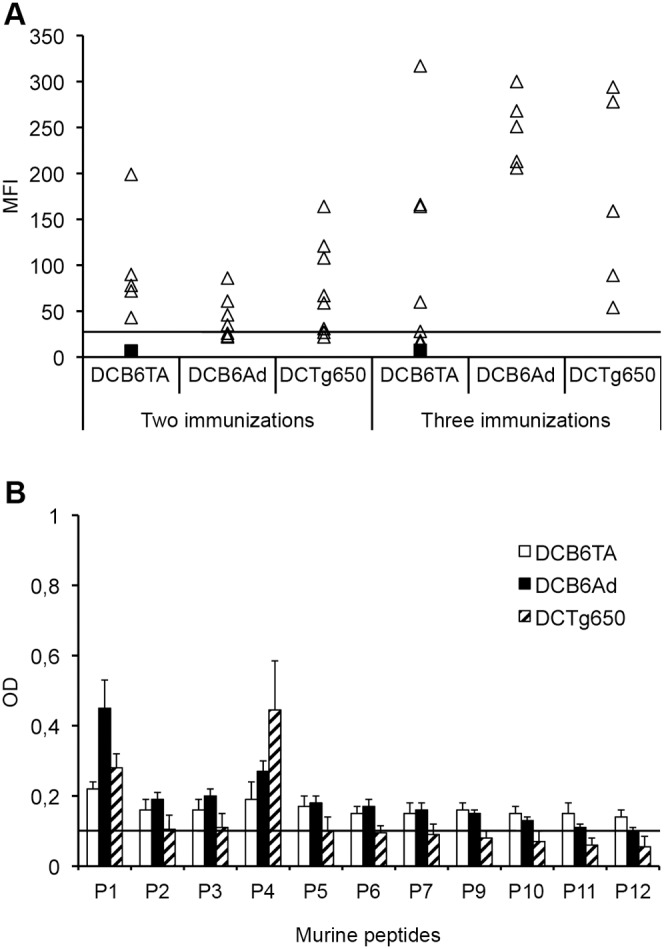
Antibody response against native PrPc elicited in C57BL/6 wt mice after DC vaccination. (A). Sera collected after two or three immunizations with DCB6AdTA, DCB6AdhPrP or DCTg650AdTA were tested (1/50 dilution) for their capacity to bind mPrP expressed on activated EL4 cells. The level of serum binding was expressed as MFI revealed by incubation with a biotinylated anti-Ig antibody followed by APC-conjugated streptavidin. Fluorescence was analysed by flow cytometry. Each point represents the MFI obtained for an individual serum sample from immunized (triangle) or unimmunized (black square) mice. (B). Epitope mapping of serum antibodies generated after immunization. Individual immune sera (1/50 dilution) were tested by ELISA for binding to each 30-mer mPrP peptides (10 µg/well) coated on plastic microplates. Each bar is the mean OD±SE of five or four sera from mice immunized with DCB6AdTA, DCB6AdhPrP or DCTg650AdTA, respectively.

Mice were immunized twice with 5×10^5^ DCs and sera were collected 2 weeks later. Sera from the three groups collected after two immunizations, including mice immunized with DCB6AdTA, stained EL4 murine cells (Fig. 5A). Mice were challenged i.p. with the 0.5% (w/v) 139A brain homogenate and a third DC injection was given 3 weeks later. Serum binding to EL4 cells was enhanced in all three groups (Fig. 5A). However, the level of antibodies to endogenous mPrP was higher and more homogeneous in mice immunized with DCB6AdhPrP (mean fluorecence intensity (MFI): 248±39) than with DCB6AdTA (mean MFI: 110±112), with intermediate values being observed for DCTg650-treated mice (mean MFI:175±108), suggesting that mPrP-specific responses developed in wt mice. Linear epitope mapping showed that antibodies produced in DCB6AdhPrP-treated mice weakly recognized P1 and P4 but that no reactivity for PrP peptides was detected in DCB6AdTA-treated mice. (Fig. 5B).

**Figure 6:**
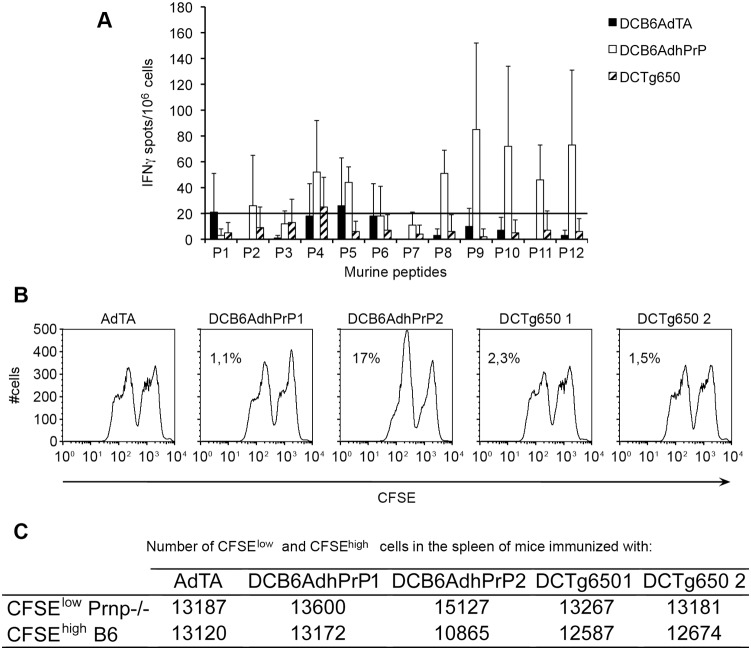
T cell responses in DC-vaccinated C57BL/6 wt mice. (A). Frequency of IFN-γ secreting T cells in the spleens of immunized C57BL/6 B6 mice evaluated by ELISPOT assay. Spleen cells (10^6^/well) from mice receiving DCB6AdTA, DCB6AdhPrP or DCTg650AdTA were stimulated 18 h at 37°C with each 30-mer peptides from mPrP at a concentration of 10 µg/ml. The frequency of peptide-specific IFN-γ secreting T cells was calculated after subtracting the mean number of spots of triplicate cultures obtained in the absence of peptide. Each column shows the mean numbers of spots±SE per 5×10^5^ spleen cells from two, four or six mice that received DCB6AdTA, DCB6AdhPrP or DCTg650AdTA, respectively. (B). In vivo cytotoxic activity was measured by flow cytometry: spleen cells from mice vaccinated with DCB6AdTA, DCB6AdhPrP or DCTg650AdTA were collected 20 h after injection of 10^7^ CFSE^low^ *Prnp^−/−^* cells mixed with 10^7^ CFSE^high^ cells expressing mPrP (B6). (C). Number of CFSE^low^ and CFSE^high^ cells in the spleen of vaccinated or not individual mice and percentage of specific lysis of PrP^+^ over *Prnp^−/−^* splenocytes calculated as detailed in “Materials and Methods”.

Spleen cells from mice immunized with DCB6AdhPrP secreted IFNγ in response to a broad spectrum of murine peptides while mice treated with the DCTg650AdTA or DCB6AdTA developed no significant response (Fig. 6A).

In vivo cytotoxicity was measured in immunized mice after injection of B6 CFSE*^high^* cells mixed with *Prnp^−/−^* CFSE *^low^*cells. A low antigen-specific cytotoxicity (17%) for cells expressing mPrP was observed in one of two mice immunized with DCB6AhPrP, but not in mice immunized with DCB6AdTA or DCTg650AdTA (Fig. 6B).

**Figure 7:**
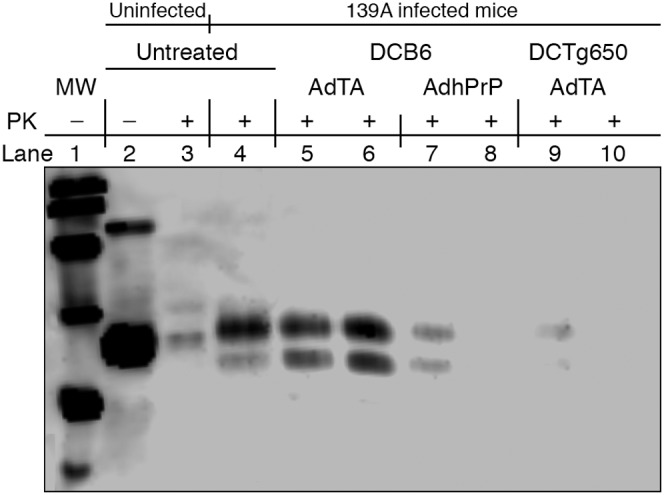
Measurement of PK-resistant PrPres in untreated and DCB6AdTA-, DCB6AdhPrP- or DCTg650AdTA-vaccinated C57BL/6 wt mice. Western blotting was performed on PK-treated PTA precipitates prepared from 10 mg of spleens collected at 10 weeks after challenge with 0.5% 139A scrapie. Samples were digested with 50 µg/ml PK, heated at 100°C and loaded on 12.5% polyacrylamide/SDS gel. Blots were incubated with 1/5000 diluted SAF84 (Anti-mPrP mAb) and revealed with peroxidase-conjugated goat anti-mouse Ig antibody. Lane 1 = Molecular weight. Lane 2 = normal spleen. Lane 3 = normal spleen+PK. Lane 4 = 139A-infected spleen+PK. Lanes 5–6 = DCB6TA-treated infected spleen+PK. Lanes 7–8 = DCB6AdPrP-treated infected spleen+PK. Lanes 9–10 = DCTg650AdTA-treated infected spleen+PK.

PrPres was measured by Western blot in the spleens of two mice randomly chosen within each group at 10 weeks after infection with the 0.5% suspension of 139A scrapie (Fig. 7). The amount of PrPres in mice immunized with DCB6AdTA was similar to that observed in untreated 139A mice (Lanes 5–6 vs Lane 4) while mice that received DCB6AdhPrP or DCTg650AdTA mice had strongly reduced levels of PrPres (Lanes 7–10).

Significant prolongation of both incubation and survival times were observed in all vaccinated groups. The best result was achieved after immunization with DCTg650AdTA (n = 5) resulting in a 37 day increase in survival (p = 0.0003) and in one mouse remaining free of disease with no PrPres in its spleen at 300 dpi (Table 2C). Mice immunized with DCB6AdPrP or DCB6AdTA survived significantly longer than untreated mice (225±12, p = 0.028 and 223±16, p = 0.008 versus 209±8 dpi, respectively; Table 2C) with one mouse surviving in the DCB6AdTA group without clinical signs of disease and no PrPres in the spleen at 300 days. The duration of the clinical stage was not significantly modified by the three treatments. Protection afforded by DCB6AdPrP was not significantly better than that afforded by DCB6AdTA, but vaccination with DCTg650AdTA prolonged the incubation time (p<0.01) and the survival time (p = 0.06) compared to DCB6AdTA and DCB6AdPrP-treated groups.

### T cell infiltration in the brains of vaccinated mice by immunohistochemical analysis

To check for possible T cell infiltration, brains were collected at 10 weeks dpi and at the terminal stage of the disease. Paraffin-embedded tissue sections collected at 10 weeks were stained with H&E and no signs of inflammation were detected (data not shown). Brains collected at the terminal stage were stained with anti-CD3 mAb by indirect immunofluorescence. Table 3 summarizes the mean numbers of CD3^+^ cells counted in the different regions of the brain of two mice per group. A small number of CD3^+^ T cells infiltrated all regions of brains but no obvious differences were observed whatever mice were vaccinated or not.

**Table 3:** Immunohistochemical analysis of T cell infiltration in the brains of untreated or vaccinated mice.

Regions	Mean number (±SD) of CD3^+^ T cells infiltrating brains of 139A infected mice					
	Untreated	AdTA	AdhPrP	DCB6AdTA	DCB6AdhPrP	DCTg650
Anterior	2.45±0.07	3.24±0.7	4,12±1.86	2.74±0.1	2.83±0.42	3.63±1.06
Median	4.12±0.17	4.45±0.78	6.66±0.17	4.50±0	5.67±0.19	4.05±1.87
Posterior	3.25±1.76	3.19±0.73	2.66±0.01	1.95±0.15	4.22±1.35	2.63±0.35

Mean number of CD3^+^ infiltrating/brain area.

## Discussion

This study is based on an original methodology, in which a xenogeneic antigen, hPrP, is presented either directly by transgenic DCs together with adenovirus danger signals or via adenoviral transduction. Using an adenoviral vector was based on its known efficiency at eliciting CD8^+^ T cells [35]–[36] and antibodies [37]–[38] against self proteins.

Prime-boost immunization was performed by first injecting the recombinant AdhPrP and then boosting with the PrP plasmid, since other prime-boost regimens including AdhPrP-AdhPrP, phPrP-AdhPrP (data not shown) or PcDNA encoding mouse PrP [22]–[23] were unable to induce high-titer antibodies in C57BL/6 wt mice. In this study, *Prnp^−/−^* mice were immunized with AdhPrP in parallel with wt mice to validate the Ad vectors and immunization protocol in the absence of PrP tolerance. AdhPrP was capable of raising antibodies specific for native mPrP in *Prnp^−/−^* mice but only poorly in wt mice. Linear peptide mapping identified in the sera of *Prnp^−/−^* mice antibodies specific for several B-cell epitopes located in the N-terminal unstructured portion of mPrP but only one located between aa 83 and 112 (P4) in the sera of wt mice. These data are in agreement with those obtained with *Prnp^−/−^* mice immunized with a PrP plasmid and confirmed that wt mice have tolerized at least a part of the B cell repertoire recognizing cell surface exposed epitopes [23].

The results were clearly different with DC vaccines that have the capacity to break tolerance against a variety of self antigens [36]–[39]. In our hands, BM-derived CD11^+^ DCs from C57BL/6 mice transduced with AdhPrP or AdTA, as well as DCs from Tg650 mice transduced with AdTA, were much more potent in generating antibodies to native mPrP than direct immunization with AdhPrP. However, almost no or few murine PrP peptides (P1/P4) were recognized, suggesting that antibodies may bind to conformational epitopes not detectable by linear peptide ELISA. This high production of antibodies specific for mPrP may be related to the maturation state of DCs induced by LPS and Ad vector transduction, which up-regulate MHC class II and B7.2 costimulatory molecules as shown in this study and also mPrP as reported earlier [40]. During DCB6AdhPrP vaccination of B6 mice, an increased presentation of murine and human surface PrP coexpressed on the same DC, together with exogenous help directed against adenoviral proteins (attested by the presence of antibodies against the hexon and fiber Ad proteins in the sera), may have stimulated antibody production to self-PrP. High levels of antibodies to self-mPrP were also elicited in 3 out of 6 recipients receiving DCs from B6 mice transduced with the transactivator Ad not expressing hPrP transgene (AdTA). In this situation, breaking tolerance was probably achieved when matured DCs copresented viral epitopes to CD4^+^ Th cell and up-regulated surface mPrP to B cells. Yet, the mechanism might be different in the course of vaccination with DCTg650, since DCs express hPrP in the m*Prnp^−/−^* context. It has been reported that DCs transduced with Ad vectors produced type I IFN, which promoted the maturation of both transduced and bystander DCs [41].

Direct AdhPrP vaccination protocol did not fully overcome anergy, and was also inefficient at providing a good level of protection against prion challenge. On the contrary, mice immunized with DCTg650AdTA succumbed at 246 dpi; this delay corresponded to the mean survival time of mice infected with only 0.01% brain homogenate (244 dpi), as calculated from a 139A dose-effect study. These data clearly demonstrated that the immune response to PrP after DC vaccination can inhibit PrP^Sc^ replication in a very efficient manner as demonstrated by the nearly absence of early accumulation of PrP^Sc^ in the spleen of DCTg650AdTA-treated mice.

Compared to the multiple trials that have been undertaken to actively generate systemic immune responses specific for PrP in wt mice, our results show the best protection. Indeed, many immunization strategies using DNA containing foreign helper T-cell epitopes [42], repeated injection of xenogenic PrP peptides conjugated to a carrier protein [43], peptides formulated in an adjuvant derived from Mycobacterium [44] or multiple injection of recombinant bovine PrP in complete Freund's adjuvant [27] largely failed to induce high levels of antibodies directed against the native PrP and afforded modest increases in the average survival time. Only mucosal immunization was able to induce protection against a challenge delivered by the oral route, probably linked to the induction of PrP antibodies at the mucosal surfaces [45]–[48].

In light of these results, we tried to establish a relation between the protection and the type or magnitude of anti-mPrP humoral responses generated by different protocols of vaccination. Titers of auto-antibodies that bind to mPrP-expressing cells were much higher after DC vaccination compared to direct immunization with AdhPrP. However, the reactivity of antibodies with linear peptides in ELISA was low, suggesting that most of them recognized conformational epitopes on mPrP molecule and therefore might be efficient at blocking PrP^Sc^ replication. In these trials, several observations suggest that the level of antibody binding to membrane PrP^c^ is related to the level of protection: mice displaying the highest antibody level either survived (DCAdTA-treated mice) or had the longest survival (AdhPrP-treated mice). It is noteworthy that while the evaluation of the antibody level was not truly quantitative, it did allow comparison of the intensity of binding of all serum samples, since results presented were obtained in the same assay for all experiments. Nevertheless, the binding capacity of antibodies on whole PrP^c^ does not necessary parrallel the neutralizing activity, which could implicate specific epitopes, and thus not predictive of in vivo protection.

Regarding the safety of prion vaccines, very few studies have concerned the identification or generation of CD8^+^ CTLs against self-PrP^c^ and thus their possible impact in prion disease or inducing deleterious autoimmunity. In two published vaccination trials, PrP-specific CD4^+^ and CD8^+^ T cells were induced as determined by detection of intracellular IFNγ [49]–[50]. The treatment dramatically delayed the onset of prion disease when given intracerebrally, although after the first symptoms appeared, it progressed rapidly. The authors attributed the deleterious effect to antibody-mediated neuronal damage but did not analyse the possible contribution of PrP-specific CTLs [49]. In the present study, direct AdhPrP immunization induced a variable frequency of precursor T cells secreting IFNγ in response to P8 that contains the major CD4^+^ epitope 155–170 of mPrP [21]–[22]. The weakness of T cell responses could be explained by tolerance to mPrP and/or by preferential priming of Th cells directed to viral capsid proteins. This immunization procedure induced a significant in vivo cytotoxicity for target cells overexpressing hPrP but not for cells normally expressing mPrP. The lack of cytotoxicity for self-PrP may be due either to an absence of murine MHC class I epitopes shared between mPrP and hPrP, or to a low TCR affinity of these cells which was insufficient for lysis of target cells expressing a physiological level of mPrP. Furthermore, we may not be certain that CD8^+^ T cells were responsible for the in vivo lysis of CFSE-labeled targets, since effectors were not identified in this assay. Adoptive transfer experiments are currently done to answer these questions. In contrast, DC vaccine strategy did not generate significant T cell responses specific for mPrP. Some CD3^+^ T cells were found in the brain of 139A-infected mice at the terminal stage of the disease but similar numbers were detected whether or not mice were vaccinated. Similarly, it has been reported that in the absence of previous vaccination, CD8^+^ T cells specific for PrP, without CTL activity, infiltrate the brain of intracranially infected mice [51]. Collectively, the in vivo and in vitro data strongly suggested that cytotoxic cells did not participate in the partial protection demonstrated here, and did not induce inflammatory reactions in the brain.

In conclusion, our results show that DCs were very potent at breaking tolerance against endogenous mPrP and favor the production of high titered antibodies against its native conformation over T-cell responses resulting in substantial delay in disease progression without triggering autoimmune reactions. The underlying mechanism may be related to a high expression of membrane mPrP on mature DCs temporally coupled with the generation of a strong helper response elicited by viral vector antigens. During the past years, numerous clinical trials have been carried out to assess the safety and efficacy of DCs-based cancer vaccines for a broad range of malignant diseases and potent anti-tumor immune responses together with the absence of toxicity and autoimmunity were recorded [52]. The high levels of antibody and absence of autoimmunity observed in our work make DC vaccines a promising therapeutic strategy that might be applied in neurodegenerative diseases such as Alzheimer.

## Materials and Methods

### Ethics statement

All animals were handled in strict accordance with good animal practice as defined by the relevant national and/or local animal welfare bodies, and all animal work was approved by the local ethics committee of ENVA (Ecole Vétérinaire d'Alfort).

### Mice

C57BL/6 mice from Charles Rivers and *Prnp^−/−^* mice knock out for the murine *Prnp* gene on a C57BL/6 background (donated by Dr. Charles Weissmann, Institut of Neurology, Medical Research Concil Prion unit, London, UK) were used for immunization and as recipients for cell transfer experiments. For preparation of DCs, Tg650 transgenic mice overexpressing hPrP with methionine at codon 129 on a mouse *Prnp^−/−^* background [53] were kindly provided by Hubert Laude (Jouy en Josas, France).

### Vectors, immunisation protocols and prion challenge of mice

An adenovirus type 5 (AdTRMet) expressing the hPrP has been previously described [34]. The PrP gene is under the control of the tetracycline-responsive promoter, allowing tight regulation of PrP expression. The AdCMNtTA (AdTA) virus encoding the transactivator of the tetracycline-regulated system, under the control of the CMV promoter and in fusion with a nuclear localisation signal, was kindly provided by Dr. H. Hamada (Cancer Institute, Tokyo, Japan). Immunization of C57BL6 mice or *Prnp^−/−^* mice was conducted by inoculation of a mixture of equal doses of each virus (AdTRMet /AdCMNtTA and referred as AdhPrP) by the intramuscular route. The mixture was always prepared immediately prior to inoculation of mice. This immunization strategy was chosen at the request of a French regulatory body, as a mean of diminishing the risk of bystander gene transfer by a vector expressing hPrP at a high level. A boost was administered 3 weeks later by intramuscular inoculation of 50 µg of pAdTRMet [34], a plasmid harbouring the same PrP transcription unit as AdTRMet together with 50 µg of pTA encoding the transactivator of the tetracycline-regulated system (Clontech, Palo Alto, USA). The mix of the two plasmids was referred as phPrP. Control mice were inoculated with AdTA or pTA. Another immunization protocol used DCs prepared as described below. DCs from C57BL/6 mice were transduced with AdTA (DCB6AdTA) or AdTA/AdhPrP (DCB6AdhPrP) and DCs from Tg650 mice were transduced with AdTA alone (Tg650AdTA). C57BL/6 mice received 5×10^5^ DCs intraperitoneally at weeks 0 and 3. Immunization was repeated once three weeks after prion challenge. All experiments were conducted according to the rules of the local ethical committee of the Alfort veterinary school. All animals were identified individually by a transponder. Mice were challenged two weeks after the second immunization by the intraperitoneal route with 100 µl of 0.5% homogenate prepared from terminal 139A-scrapie brain, corresponding to 104.33 ID50 [54]. The dosage of 0.05% and 0.01% 139A brain homogenate were assessed in survival experiments. The progression of the disease was monitored by observing twice a week starting at 20 weeks post infection (p.i.), activity levels and competence on a set of parallel bars as described previously [54].

### Murine DCs and adenoviral transduction

DCs were generated from bone marrow of C57BL/6 or Tg650 mice. Cells were resuspended at 10^6^ cells/ml in RPMI 1640 supplemented with 10% heat inactivated FCS, 1000 u of recombinant GM-CSF (Peprotech, France), glutamine, sodium pyruvate HEPES, 50 µM 2-ME, penicillin and streptomycin and cultured at 37°C in a 5% CO_2_ for 3 days. Cells were provided with fresh medium containing GM-CSF, harvested 2 days later and washed. For transduction, AdhPrP was used at a multiplicity of infection of 100. DCs were matured by adding LPS at 2 µg/ml for 20 h. The transduction efficiency of the DCs was determined by flow cytometry using the anti-hPrP 4F2 mAb. Surface markers of CD11c^+^ DCs were analysed by indirect immunofluorescence using antibodies against IAb and B7-2 molecules.

### ELISPOT Assay

The number of antigen-specific IFNγ producing cells was evaluated in the spleens of immunized mice by ELISPOT assay. Briefly, nitrocellulose-based 96-well plates (Millipore) were coated with anti-mouse IFNγ capture Abs (1/500) (Becton Dickinson) for 2 h at 37°C, followed by an overnight incubation at 4°C. Plates were washed with PBS-0.05% Tween 20 (PBS-T) and blocked with medium containing 10% FCS for 2 h at 37°C. Responders from individual mice were seeded at 10^6^ cells/well for total splenocytes and stimulated with medium or 10 µg/ml of each peptides from a library of murine overlapping 30-mer PrP peptides (Table 1). Plates were incubated at 37°C in 5% CO_2_ for 24 h, washed with PBS-T, and then incubated for 2 h at 37°C with biotinylated anti-mouse IFNγ detection Abs (Becton Dickinson). After washing, alkaline phosphatase conjugated to streptavidin was added (Roche) (1/500 dilution, 100 µl/well) for 2 h. Secreting cells were visualized using tetrazolium nitroblue/bromochloro-indolylphosphate (TNB/BCIP) substrate (Promega) and spots were counted using an automatic ELISPOT plate reader (ICI). Test wells were assayed in triplicate and the frequency of peptide-specific T cells was calculated after subtracting the mean number of spots obtained in the absence of peptide. A frequency of spots below 20 was considered as non significant.

### Antibody titration (ELISA)

Plates (Maxisorp, Nunc) were coated with 10 µg/ml of murine 30-mer PrP peptides (Table 1) in 0.1 M sodium carbonate buffer overnight at 4°C, washed with PBS and blocked with 1% non-fat milk in PBS-T for 2 h at 37°C. Sera were diluted 1∶50 and added in duplicate and incubated overnight at 4°C. After washing, 200 µl of peroxidase conjugated anti-mouse Ig (1/5000) was added and left 2 h at room temperature. Plates were washed and 200 µl/well of freshly prepared H_2_O_2_/ O-phenylediamine (OPD) substrate solution (Sigma) was added. The reaction was stopped with sulfuric acid (2N) and plates were read at 492 nm.

### Flow Cytometry analysis

*Prnp^−/−^* transfected EL4 T cells expressing murine PrP (mPrP) as previously published [22] were used as target for testing serum antibodies specific binding in indirect immunofluorescence. EL4 cells were activated overnight on anti-CD3 antibody coated-plastic (2-C11, 10 µg/ml) before testing immune sera for maximal expression of PrP^c^. Non transfected wt EL4 T cells, even upon activation, express only weak levels of cell surface PrP^c^ (unpublished observations). The level of hPrP and mPrP expression was checked by indirect immunofluorescence using 4F2 and SAF83 mAb, respectively (from Dr. Grassi, CEA, France). After blocking Fc receptors with antibody 2.4G2 for 20 min at 4°C in FACS buffer, cells were incubated with control or immune sera diluted 1/10 or 1/50 for 20 min at 4°C and washed and PE-labelled anti-mouse Ig was then added for 20 min. To increase the sensitivity of serum antibody detection on EL4 cells, revelation was performed using a biotinylated rat anti-mouse IgG and streptavidin-APC (BD Biosciences) as second antibody. Sera from peptide-immunized mice were considered significantly positive for native PrP binding when the mean fluorescence intensity (MFI) was greater than the MFI+3SD obtained with sera from normal mice. The specificity of this assay for detecting anti-native PrP^c^ Ab was previously validated on non-transfected EL4 cells activated under the same conditions as transfected EL4 cells. MHC class II, B7.2 and hPrPmolecule expression was quantified at the surface of DCs before and after in vitro maturation and transduction with AdTA or AdhPrP. Anti-IAd, anti-CD86 (BD Biosciences) and anti-hPrP mAbs were incubated after Fc receptor blocking with DCs and revealed with PE-conjugated anti-mouse IgG2a mAb. After washing, cells were analysed on a FACS-calibur flow cytometer using Cell Quest software (Becton-Dickinson)

### In vivo cytotoxicity test

Cytolytic T cells were detected by in vivo clearance of CFSE-stained target cells expressing murine or human PrP or no PrP. Control target splenocytes from *Prnp^−/−^* mice were stained with 0.5 µM CFSE (CFSE^low^) (Vybrant CFDA SE Cell Tracer Kit; Molecular Probes). Antigen-specific target splenocytes from C57BL/6 (mPrP) or Tg650 (over expressing hPrP) mice were stained with 5.0 µM CFSE (CFSE^high^). Equal numbers of CFSE-labelled control and experimental target splenocytes (10×10^6^ each, mixed 1∶1) were injected *iv* into immunized recipient and control naive mice. Eighteen hours after CFSE-labelled target cell injection, splenocytes from recipients were collected, red blood cells lysed and analyzed by flow cytometry, gating on CFSE-positive splenocytes. The percentage of specific cytotoxicity was calculated using the following formula:[1−(CFSE^high^exp/(CFSE^low^exp+CFSE^high^exp)/(CFSE^high^control/(CFSE^low^control+CFSE^high^ control)]×100 in which #CFSE^high^ represents the number of antigen-specific target cells and #CFSE^low^ represents the number of control target cells recovered from either naive or immunized mice.

### Western blot analysis of PrPres

10% spleen homogenates were centrifuged 1 min at 1000 rpm. 500 µl of supernatant was mixed with an equal volume of 4% sarkosyl in PBS pH 7.4 and digested with 50 u/ml Benzonase (Benzon nuclease, purity 1; Merck) for 30 min at 37°C. Samples corresponding to 10 mg of spleen were precipitated with 0.3% sodium phosphotungstic acid (PTA) at 37°C for 30 min and pellets were resuspended in 20 µl PBS 0.1% sarkosyl. Samples were digested with 20 µg/ml proteinase K (PK) at 37°C for 15 min, blocked by addition of loading buffer (125 mM Tris pH 6.8, 20% glycerol, 4% 2-ME, 8 mM 4-(2-aminoethyl)-benzene sulfonyl fluoride, and 0.02% bromophenol blue) and heated at 100°C for 10 min. After electrophoresis in a 12.5% polyacrylamide/SDS gel, proteins were electrotransferred onto a PVDF membrane and blocked in 5% non-fat milk in 0.1% PBS-T. Blots were incubated overnight at 4°C in a 1/30000 dilution of anti-PrP mAb (SAF83), then for 1 h with peroxidase-conjugated goat anti-mouse IgG antibody, and finally proteins were revealed by chemoluminescence (ECL^+^, Amersham).

### Checking for autoimmune-related pathology

Brains were collected immediately after sacrifice and immersion-fixed in 4% para-formaldehyde. They were transversally cut into three equal, anterior, median and posterior and embedded in paraffin. Five µm-thick sections were cut on a Leica RM2145 microtome and deposited on Superfrost Menzel-Glaser slides and then air dried. Evaluation of lesions was done by a certified veterinary pathologist. Haematoxylin/eosin (H&E) staining was performed on embedded paraffin sections of brains collected 10 weeks after challenge and immunofluorescence staining on brains collected at terminal stage of the disease. To detect T cells, a 1/200 diluted anti-CD3 mAb clone SP7 (Thermo Scientific, Interchim) was incubated for 30 min and then revealed with a FITC-goat anti-rabbit IgG (Jackson, Interchim) at 1/200 dilution for 30 min. Slides were mounted in Vectashield fluorescent-mounting medium containing Dapi stain to confirm the presence of cells (Vector Laboratories, Burlingame, CA). Photomicrographs were taken with a Olympus BX61 fluorescent microscope and analysed with Gimp software. Two mice per treatment were examined and the number of CD3^+^ cells was quantified by counting five foci at 400× magnification in three sections for each brain sector.

### Statistical Analyses

Comparison of the mean incubation time, survival or duration of clinical stages was performed with the Mann-Whitney test. Mean precursor frequencies were compared using the Student's *t* test.

## Supporting Information

Data S1 Antibody response against native PrPc elicited in Prnp−/− and C57BL/6 wt mice after direct Ad immunization. Sera collected after two or three immunizations with AdTA or AdhPrP were tested (1/50 dilution) for their capacity to bind human PrP expressed on C5 cells. The level of serum binding was expressed as mean fluorescence intensity (MFI) and revealed by incubation with a biotinylated anti-Ig followed by APC-conjugated streptavidin. Fluorescence was analysed by flow cytometry. Each point represents the MFI of an individual serum sample from immunized (triangle) or unimmunized (black square) mice.
(0.05 MB TIF)
