# Supplementary figures and images for: Dendritic Cell-Mediated-Immunization with Xenogenic PrP and Adenoviral Vectors Breaks Tolerance and Prolongs Mice Survival against Experimental Scrapie
Source: PLoS One. 2009 Mar 19;4(3):e4917. doi: 10.1371/journal.pone.0004917 (PMC2654673; doi:10.1371/journal.pone.0004917)

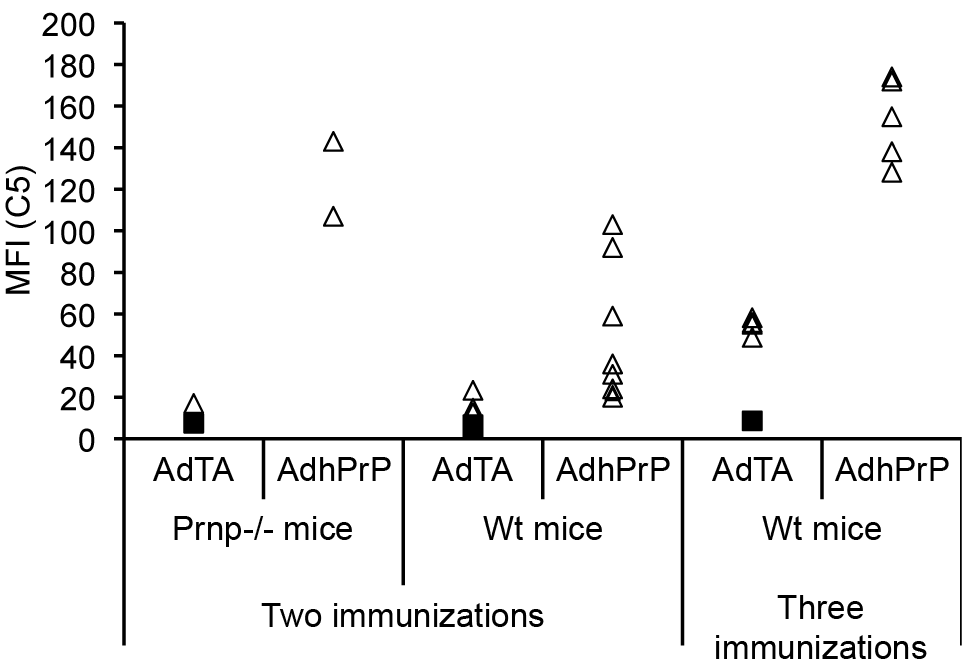

Supplement: Data S1 — Antibody response against native PrPc elicited in Prnp−/− and C57BL/6 wt mice after direct Ad immunization. Sera collected after two or three immunizations with AdTA or AdhPrP were tested (1/50 dilution) for their capacity to bind human PrP expressed on C5 cells. The level of serum binding was expressed as mean fluorescence intensity (MFI) and revealed by incubation with a biotinylated anti-Ig followed by APC-conjugated streptavidin. Fluorescence was analysed by flow cytometry. Each point represents the MFI of an individual serum sample from immunized (triangle) or unimmunized (black square) mice. (0.05 MB TIF) [file pone.0004917.s001.tif]
